# Supplementary material for: Detection of Meat Adulteration Using Spectroscopy-Based Sensors
Source: Foods. 2021 Apr 15;10(4):861. doi: 10.3390/foods10040861 (PMC8071343; doi:10.3390/foods10040861)
Supplement: Supplementary file 1 [file foods-10-00861-s001.zip › foods-1171373-supplementary.pdf]

Supplementary file

Pork - Chicken

Table S1a. Confusion matrix for SVMs classification after feature extraction using PLS components (number of LVs= 3, explained variance= 96.64%) for the external validation (*n*=30), using Vis data for fresh samples of pork/chicken and considering five classes, from 0% pork-100% chicken (0%) to 100% pork-0% chicken (100%).

	Predicted class					
True class	0%	25%	50%	75%	100%	Recall (%)
0%	2	0	4	0	0	33.33
25%	0	2	4	0	0	33.33
50%	0	0	6	0	0	100.00
75%	0	0	1	4	1	66.67
100%	0	0	1	2	3	50.00
Precision (%)	100.00	100.00	37.50	66.67	75.00	Accuracy (%) 56.67

Table S1b. Confusion matrix for SVMs classification after feature extraction using PLS components (number of LVs= 3, explained variance= 96.55%) for the external validation (*n*=30), using Vis data for fresh samples of pork/chicken and considering three classes: 0% pork-100% chicken (0%) - adulterated (A) - 100% pork-0% chicken (100%).

	Predicted class			
True class	0%	A	100%	Recall (%)
0%	6	0	0	100.00
A	0	18	0	100.00
100%	0	2	4	66.67
Precision (%)	100.00	90.00	100.00	Accuracy (%) 93.33

Table S2a. Confusion matrix for SVMs classification after feature extraction using PLS components (number of LVs= 3, explained variance= 92.53%) for the external validation (*n*=30), using Fluo data for fresh samples of pork/chicken and considering five classes, from 0% pork-100% chicken (0%) to 100% pork-0% chicken (100%).

	Predicted class					
True class	0%	25%	50%	75%	100%	Recall (%)
0%	1	5	0	0	0	16.67
25%	0	6	0	0	0	100.00
50%	0	0	5	1	0	83.33
75%	0	0	0	6	0	100.00
100%	0	0	2	4	0	0.00
Precision (%)	100.00	54.55	71.43	54.55	NaN[1]	Accuracy (%) 60.00

[1] NaN: Not a Number

Table S2b. Confusion matrix for SVMs classification after feature extraction using PLS components (number of LVs= 3, explained variance= 93.47%) for the external validation (*n*=30), using Fluo data for fresh samples of pork/chicken and considering three classes: 0% pork-100% chicken (0%) - adulterated (A) - 100% pork-0% chicken (100%).

	Predicted class			
True class	0%	A	100%	Recall (%)
0%	6	0	0	100.00
A	0	18	0	100.00
100%	0	6	0	0.00
Precision (%)	100.00	75.00	NaN[1]	Accuracy (%) 80.00

[1] NaN: Not a Number

Table S3a. Confusion matrix for SVMs classification after feature extraction using PLS components (number of LVs= 3, explained variance= 96.22%) for the external validation (*n*=30) using MSI data for fresh samples of pork/chicken, and considering five classes, from 0% pork-100% chicken (0%) to 100% pork-0% chicken (100%).

	Predicted class					
True class	0%	25%	50%	75%	100%	Recall (%)
0%	6	0	0	0	0	100.00
25%	0	6	0	0	0	100.00
50%	0	0	6	0	0	100.00
75%	0	0	0	6	0	100.00
100%	0	0	0	3	3	50.00
Precision (%)	100.00	100.00	100.00	66.67	100.00	Accuracy (%) 90.00

Table S3b. Confusion matrix for SVMs classification after feature extraction using PLS components (number of LVs= 3, explained variance= 96.23%) for the external validation (*n*=30), using MSI data for fresh samples of pork/chicken and considering three classes: 0% pork-100% chicken (0%) - adulterated (A) - 100% pork-0% chicken (100%).

	Predicted class			
True class	0%	A	100%	Recall (%)
0%	6	0	0	100.00
A	0	18	0	100.00
100%	0	0	6	100.00
Precision (%)	100.00	100.00	100.00	Accuracy (%) 100.00

Table S4a. Confusion matrix for SVMs classification after feature extraction using PLS components (number of LVs= 3, explained variance= 96.56%) for the external validation (*n*=30), using MSI data for frozen-thawed samples of pork/chicken and considering five classes, from 0% pork-100% chicken (0%) to 100% pork-0% chicken (100%).

	Predicted class					
True class	0%	25%	50%	75%	100%	Recall (%)
0%	6	0	0	0	0	100.00
25%	0	6	0	0	0	100.00
50%	0	0	5	1	0	83.33
75%	0	0	0	3	3	50.00
100%	0	0	0	0	6	100.00
Precision (%)	100.00	100.00	100.00	75.00	66.67	Accuracy (%) 86.67

Table S4b. Confusion matrix for SVMs classification after feature extraction using PLS components (number of LVs= 3, explained variance= 96.63%) for the external validation (*n*=30), using MSI data for frozen-thawed samples of pork/chicken and considering three classes: 0% pork-100% chicken (0%) - adulterated (A) - 100% pork-0% chicken (100%).

	Predicted class			
True class	0%	A	100%	Recall (%)
0%	6	0	0	100.00
A	0	16	2	88.89
100%	0	0	6	100.00
Precision (%)	100.00	100.00	75.00	Accuracy (%) 93.33

Beef - Offal

Table S5a. Confusion matrix for SVMs classification after feature extraction using PLS components (number of LVs= 3, explained variance= 97.82%) for the external validation (*n*=30), using Vis data for fresh samples of beef/bovine offal and considering five classes, from 0% beef-100% offal (0%) to 100% beef-0% offal (100%).

	Predicted class					
True class	0%	25%	50%	75%	100%	Recall (%)
0%	6	0	0	0	0	100.00
25%	0	6	0	0	0	100.00
50%	0	6	0	0	0	0.00
75%	0	1	0	5	0	83.33
100%	0	0	0	0	6	100.00
Precision (%)	100.00	46.15	[1]NaN	100.00	100.00	Accuracy (%) 76.67

[1] NaN: Not a Number

Table S5b. Confusion matrix for SVMs classification after feature extraction using PLS components (number of LVs= 3, explained variance= 98.23%) for the external validation (*n*=30), using Vis data for fresh samples of beef/bovine offal and considering three classes: 0% beef-100% offal (0%) - adulterated (A) - 100% beef-0% offal (100%).

	Predicted class			
True class	0%	A	100%	Recall (%)
0%	5	1	0	83.33
A	0	18	0	100.00
100%	0	0	6	100.00
Precision (%)	100.00	94.74	100.00	Accuracy (%) 96.67

Table S6a. Confusion matrix for SVMs classification after feature extraction using PLS components (number of LVs= 3, explained variance= 91.64%) for the external validation (*n*=30) using Fluo data for fresh samples of beef/bovine offal and considering five classes, from 0% beef-100% offal (0%) to 100% beef-0% offal (100%).

	Predicted class					
True class	0%	25%	50%	75%	100%	Recall (%)
0%	3	2	0	0	1	50.00
25%	0	6	0	0	0	100.00
50%	0	6	0	0	0	0.00
75%	0	5	1	0	0	0.0
100%	0	0	0	0	6	100.00
Precision (%)	100.00	31.58	0.00	NaN[1]	85.71	Accuracy (%) 50.00

[1] NaN: Not a Number

Table S6b. Confusion matrix for SVMs classification after feature extraction using PLS components (number of LVs= 3, explained variance= 91.38%) for the external validation (*n*=30), using Fluo data for fresh samples of beef/bovine offal and considering three classes: 0% beef-100% offal (0%) - adulterated (A) - 100% beef-0% offal (100%).

	Predicted class			
True class	0%	A	100%	Recall (%)
0%	3	3	0	50.00
A	8	10	0	55.56
100%	0	0	6	100.00
Precision (%)	27.27	76.92	100.00	Accuracy (%) 63.33

Table S7a. Confusion matrix for SVMs classification after feature extraction using PLS components (number of LVs= 2, explained variance=97.03%) for the external validation (*n*=30), using MSI data for fresh samples of beef/bovine offal and considering five classes, from 0% beef-100% offal (0%) to 100% beef-0% offal (100%).

	Predicted class					
True class	0%	25%	50%	75%	100%	Recall (%)
0%	6	0	0	0	0	100.00
25%	0	6	0	0	0	100.00
50%	0	0	6	0	0	100.00
75%	0	0	0	6	0	100.00
100%	0	0	0	0	6	100.00
Precision (%)	100.00	100.00	100.00	100.00	100.00	Accuracy (%) 100.00

Table S7b. Confusion matrix for SVMs classification after feature extraction using PLS components (number of LVs= 2, explained variance=97.11%) for the external validation (*n*=30), using MSI data for fresh samples of beef/bovine offal and considering three classes: 0% beef-100% offal (0%) - adulterated (A) - 100% beef-0% offal (100%).

	Predicted class			
True class	0%	A	100%	Recall (%)
0%	6	0	0	100.00
A	0	18	0	100.00
100%	0	2	4	66.67
Precision (%)	100.00	90.00	100.00	Accuracy (%) 93.33

Table S8a. Confusion matrix for SVMs classification after feature extraction using PLS components (number of LVs= 3, explained variance= 98.81%) for the external validation (*n*=30), using MSI data for frozen-thawed samples of beef/bovine offal and considering five classes, from 0% beef-100% offal (0%) to 100% beef-0% offal (100%).

	Predicted class					
True class	0%	25%	50%	75%	100%	Recall (%)
0%	6	0	0	0	0	100.00
25%	0	6	0	0	0	100.00
50%	0	0	6	0	0	100.00
75%	0	0	0	6	0	100.00
100%	0	0	0	0	6	100.00
Precision (%)	100.00	100.00	100.00	100.00	100.00	Accuracy (%) 100.00

Table S8b. Confusion matrix for SVMs classification after feature extraction using PLS components (number of LVs= 3, explained variance= 98.50%) for the external validation (*n* = 30), using MSI data for frozen-thawed samples of beef/bovine offal and considering three classes: 0% beef-100% offal (0%) - adulterated (A) - 100% beef-0% offal (100%).

	Predicted class			
True class	0%	A	100%	Recall (%)
0%	6	0	0	100.00
A	0	18	0	100.00
100%	0	0	6	100.00
Precision (%)	100.00	100.0	100.00	Accuracy (%) 100.00
